# Effects of Vegetation Restoration Type on Soil Greenhouse Gas Emissions and Associated Microbial Regulation on the Loess Plateau

**DOI:** 10.1002/ece3.70688

**Published:** 2024-12-23

**Authors:** Jihai Zhou, Daokun Liu, Shangqi Xu, Xiaoping Li, Jiyong Zheng, Fengpeng Han, Shoubiao Zhou, Meng Na

**Affiliations:** ^1^ Collaborative Innovation Center of Recovery and Reconstruction of Degraded Ecosystem in Wanjiang Basin co‐Founded by Anhui Province and Ministry of Education, School of Ecology and Environment Anhui Normal University Wuhu China; ^2^ Collaborative Innovation Center of Southern Modern Forestry Nanjing Forestry University Nanjing China; ^3^ Forestry Technology Center of Wuhu City Wuhu China; ^4^ State Key Laboratory of Soil Erosion and Dryland Farming on the Loess Plateau, Institute of Soil and Water Conservation Northwest A&F University Yangling Shaanxi China

**Keywords:** Loess Plateau, soil greenhouse gas, soil microorganisms, vegetation restoration

## Abstract

Investigating responses of soil greenhouse gas (GHG) emissions to vegetation restoration is important for global warming mitigation. On the Loess Plateau, a wide range of vegetation restoration strategies have been implemented to control land degradation. However, the thorough quantification of soil GHG emissions triggered by different modes of vegetation restoration is insufficient. There is still a knowledge gap regarding the regulation of soil biochemical and microbial processing on soil GHG emissions. To do so, we compared responses of soil GHG emissions to various types of vegetation restoration on the Loess Plateau, and investigated the changes in soil properties as well as microbial composition and activities. We found that artificial plantation of *Caragana korshinskii* had low soil carbon dioxide (CO_2_) emission, while natural grassland had high CO_2_ emission. The possible explanations could be related to higher moisture and microbial biomass carbon, and greater nitrogen limitation in natural grassland, which was controlled by actinomycetes and gram‐negative bacteria. Natural grassland had low soil nitrous oxide (N_2_O) emission and high methane (CH_4_) uptake, whereas *Prunus mume* had high N_2_O emission and *Medicago sativa* had low CH_4_ uptake, respectively. Soil N_2_O emission could be driven by fungi and gram‐positive bacteria which were affected by N availability and dissolved organic carbon. Soil CH_4_ consumption was associated with anaerobic bacteria and gram‐negative bacteria which were affected by N availability and moisture. These different emissions of CO_2_, N_2_O and CH_4_ generated the largest total GHG emissions for plantation of *Prunus mume* , but the smallest total GHG emissions for natural grassland and plantation of leguminous *Caragana korshinskii* . Overall, our findings suggested that the restoration of natural grassland and artificial N‐fixing shrubland like *Caragana korshinskii* should be encouraged to alleviate GHG emissions, with the practical implications for selecting suitable modes and species to improve ecological sustainability in degraded lands.

## Graphical abstract

Greenhouse gas (GHG) emissions from soils play a significant role in contributing to global warming. Vegetation types especially affect soil GHG production. Our findings suggest that the restoration of natural grassland and artificial N‐fixing shrubland like *Caragana korshinskii* should be encouraged to alleviate GHG emissions, with the practical implications for selecting suitable modes and species to improve ecological sustainability in degraded lands.

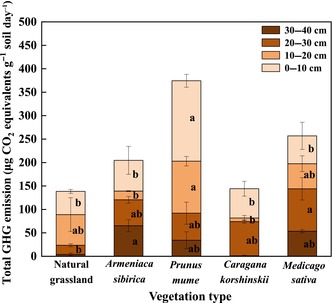

## 1 Introduction

The production of greenhouse gas (GHG), principally carbon dioxide (CO_2_), methane (CH_4_) and nitrous oxide (N_2_O) from terrestrial ecosystems, has been recognized for playing a key role in contributing to global warming (Wagner et al. 2019). Soil is a primary source or sink for GHG, where approximately 20% of CO_2_ emissions, 30% of CH_4_ emission, and 70% of N_2_O emissions to the global atmosphere originate from soils (Smith et al. 2003; Lubbers et al. 2013). It is well‐known that soil GHG production is the consequence of various biochemical processes, such as CO_2_ emission through soil respiration (Rastogi et al. 2002), N_2_O emission through mostly nitrification and denitrification (Wrage et al. 2001), and CH_4_ emission determined by the balance between methanogenesis and CH_4_ oxidation (Le Mer and Roger 2001). Thus, any small changes in soil environments that alter carbon (C) and nitrogen (N) turnover may affect its function of producing and consuming GHG (Oertel et al. 2016). In the plant–soil ecosystem, variations in vegetation communities can be a primary determinant that leads to changes in soil properties and microbial activities, via rhizosphere exudation, litter decomposition, and physiological characteristics of species (Sokol and Bradford 2019; Yang et al. 2022; Xu et al. 2022). The differences in vegetation types can therefore influence soil GHG process.

Over past decades, ecological restoration practices have expanded globally to restrain land degradation (Borchard et al. 2017; Lu et al. 2018). For instance, China has undertaken several national key ecological restoration projects since 1970s (Ouyang et al. 2016), of which the “Grain for Green” Program that croplands are converted to grasslands, shrublands and forests, is the largest (Shao et al. 2000; Lu et al. 2018). Studies have indicated that implementations of ecological restoration can enhance soil C sequestration (Deng et al. 2019; Zhang et al. 2023), but some challenges have been arising since the goal of net zero GHG emissions is widely encouraged to limit global temperature increase (Tanaka and O'Neill 2018). This calls for the need to select more appropriate modes of vegetation restoration in ecological restoration projects, to further maximize GHG emission cut. Many studies have attempted to investigate the effects of land use types on soil GHG emissions, but the results are inconsistent (Han and Zhu 2020; Chen et al. 2021; Feng et al. 2022). For example, some studies have found that restoration of natural vegetation is superior to the artificial vegetation for the improvement of multiple ecological functions in the degraded systems, mitigating soil C and N loss (Hu et al. 2020; Zhang et al. 2022; Zhou et al. 2023). A global meta‐analysis has reported that artificial plantation decreases soil CO_2_ emissions, but increases CH_4_ and N_2_O emissions compared to natural grassland (Feng et al. 2022). Han and Zhu (2020) has found that artificial forest and grassland increase soil CH_4_ efflux, but have no effect on soil N_2_O efflux, compared to natural forest. Other studies have also observed distinct responses of GHG emissions to vegetation restoration at different soil layers (Wang et al. 2023; Button et al. 2023). These various findings highlight the complex impact of vegetation types on soil GHG production, which is temporally and spatially heterogenous. As such, climate change control during ecological restoration may face challenges because of the contrasting responses of different types of GHG to the same land use. Yet, few studies have regarded a thorough quantification of all the soil GHG emissions triggered by different modes of vegetation restoration, and evaluated the comprehensive effect of vegetation restoration on soil GHG production in fragile systems.

Bacteria and fungi are primary drivers involved in C and N cycling in plant–soil ecosystems, regulating soil GHG emissions (Espenberg et al. 2024). Studies have identified that vegetation restoration along with variations in soil biogeochemical processes can alter microbial metabolic function, causing different GHG responses (Chen et al. 2021; Zhang et al. 2024). It has been reported that afforestation on the Loess Plateau results in microbial N or phosphorus (P) limitation, improving microbial demands for nutrients from SOM and consequent CO_2_ emission (Zhang et al. 2024). Chen et al. (2021) has concluded that shrubland has higher DOC content than undisturbed lands which provides sufficient available substrates for nitrifiers, benefiting for soil N_2_O emission. Meanwhile, soil moisture and temperature have been found to be largely associated with microbial‐controlled GHG emissions (Feng et al. 2022; Kong et al. 2022). Yet, it is poorly studied how microorganisms interact with vegetation and soil biochemical characteristics, and how their interactions affect the resulting GHG production in degraded lands.

The Loess Plateau is located in both arid and semiarid areas of China, and regarded as one of the most vulnerable ecosystems in the world, suffering severe soil erosion (Deng et al. 2019). Thus, the Loess Plateau is a priority region for the “Grain for Green” Program (Zhou et al. 2012). However, although some studies have evaluated GHG emissions from different ecosystems in the Loess Plateau (Ran et al. 2021; Li et al. 2023), few studies have assessed and compared GHG production consisted of CO_2_, N_2_O and CH_4_ throughout the soil profile between different types of vegetation restoration. It is still uncertain whether microbial responses to vegetation restoration are responsible for soil GHG emissions in degraded lands. These limitations resulted in challenges in optimizing restoration approaches in fragile systems. In this study, we investigated how natural vegetation restoration and artificial vegetation restoration influenced GHG emissions, soil biochemical properties and microbial communities at various soil depths in the Loess Plateau. Our objectives were to (i) compare soil GHG emissions between different modes of vegetation restoration; (ii) explore how changes in soil biochemical and microbial processes induced by vegetation restoration affect soil GHG emissions; (iii) select the optimal strategy of vegetation restoration for soil GHG mitigation. We tested the hypothesis that the restoration of natural vegetation would be better for soil GHG mitigation than the artificial vegetation on the Loess Plateau.

## 2 Material and Methods

### 2.1 Study Sites and Soil Sampling

The study was conducted in the Shanghuang village of Guyuan, located in the hilly‐gully region of Loess Plateau, China (35°59′–36°02′ N, 106°26′–106°30′ E). The site was situated from 1530 to 1822 m above sea level, occupying a semiarid area of 8.19 ha. The zonal soil was classified as *Entisols* (Chinese Soil Taxonomy 2001), containing 18.53% clay, 31.61% silt, and 49.86% sand (Wang et al. 2020b). This region had a semiarid temperate monsoon climate, with the mean annual temperature of 6.9°C. The annual precipitation was 488 mm, and the annual potential evaporation was 1669 mm. There was more than 70% precipitation occurring in the form of heavy rainstorms during the period from June to September, accompanied by the local drought and flooding, thereby leading to an increase in soil erosion. The water table was 50 m below the land surface due to the thick profile (Zhang et al. 2023). The site was characterized by the low vegetation coverage, broken topography and soil erosion due to excessive cultivation over the past decades (Wang et al. 2020b). As such, different measures of vegetation restoration such as natural restoration and artificial restoration, had been conducted under the “Grain for Green” project (Wang et al. 2015). The typical natural vegetation was grassland including *Stipa bungeana*, *Artemisia scoparia*, and *Artemisia stelleriana* . The major species composition of understory vegetation in the plantation included *Stipa bungeana*, *Lespedeza davurica*, and *Heteropappus altaicus* .

We selected five types of plant species for natural and artificial vegetation restoration, including natural grassland, artificial plantation of *Armeniaca sibirica*, artificial plantation of *Prunus mume* , artificial plantation of *Caragana korshinskii* , and artificial pasture of *Medicago sativa* . The selection of these five types of vegetation were made because they were common species that were able to thrive on the Loess Plateau, which had high potentials for ecological restoration (Chai et al. 2019). *Armeniaca sibirica* (family: *Rosaceae*) was a deciduous tree with high cold and drought resistance, which was widely distributed in northern China (Zhang et al. 2018; Wu et al. 2022). Its seed kernels had high value for food, medicine and industry (Wu et al. 2022). *Prunus mume* (family: *Rosaceae*), known as its flower, was an important ornamental plant with a cultivation history of more than 3000 years in China, characterized by strong tolerance to cold and disease as well as high adaption to poor soils (Wang et al. 2024). *Caragana korshinskii* (family: *Leguminosae*) was a perennial leguminous shrub with high drought tolerance, rapid growth and N‐fixing capacity, which was prevalently planted in arid and semiarid areas due to its great ecological function (Chai et al. 2019). *Medicago sativa* (family: *Leguminosae*) belonged to herbaceous perennial legumes, playing a key role in improving soil water preservation and soil fertility in the dryland (Yang et al. 2022). In natural grassland, the dominant species were *Stipa bungeana, Stipa grandis, Artemisia scoparia, Artemisia stelleriana, Thymus mongolicus*, and *Potentilla chinensis*. The distance among five vegetation types was less than 1 km, ensuring the similarity in microclimate. The experiment was carried out in 10 × 10 m plots and had three replicates for each vegetation type. Within each plot, a soil profile (40 cm) was dug using a cylindrical auger of 10 cm diameter. Five soil cores were randomly collected at the layer of 0–10, 10–20, 20–30, and 30–40 cm for evaluating effects of soil depth, since soil GHG production was often depth‐dependent (Wang et al. 2023; Button et al. 2023). The soil was sampled according to the diagonal five‐point method where four sampling points were selected at each end of an “X” and one point was chosen at the intersection. These five cores from the same plot were mixed to form a homogenous composite sample that was sieved through < 2 mm mesh to remove stones and visible plant residues. After that, one set of fresh soil samples was stored at 4°C for less than 1 week before subsequent analyses for GHG emissions, soil water content (SWC), microbial biomass carbon (MBC), dissolved organic carbon (DOC) and total dissolved nitrogen (TDN), as well as microbial composition and enzyme activity. Another set of soil samples was air‐dried and stored in a cool and ventilated room for the measurement of soil organic carbon (SOC).

### 2.2 Soil Characteristics

SWC was estimated gravimetrically with oven drying at 105°C for 24 h, which was regarded as the standard method due to its rapidity and accuracy (Na et al. 2022). SOC content was measured using the potassium dichromate oxidation method, by which it achieved 100% recovery, indicating a high precision (Meibus 1960). MBC was determined by chloroform‐fumigation extraction (Vance et al. 1987). Briefly, one of two subsamples (ca. 5.0 g) was fumigated using ethanol‐free chloroform in a sealed desiccator for 24 h, after which fumigated and non‐fumigated samples were extracted with 20 mL 0.5 M K_2_SO_4_ solution. The contents of C and N in non‐fumigated samples were regarded as DOC and TDN. Extracts were measured using a TOC/TN analyzer for soil MBC. The content of MBC was the difference between extractable C in fumigated and non‐fumigated samples, which was corrected by an extraction efficiency coefficient value of 0.45 for MBC (Wu et al. 1990). This chloroform‐fumigation extraction method had been calibrated by adding living bacteria and fungi to soil and extracting in the same way (Vance et al. 1987). The metabolic quotient (*q*CO_2_) was estimated by dividing soil respiration by MBC (μg CO_2_ g^−1^ soil day^−1^).

### 2.3 GHG Measurements

A sample of 100 g fresh soil was weighted into 250 mL conical flasks and the headspace was purged with pressurized air before the flask was closed with airtight rubber stopper. Flasks were incubated for 24 h without light at 25°C. After incubation, the headspace gas in the flask (6 mL) was sampled using a gas tight syringe, for subsequent measurements of CO_2_, N_2_O, and CH_4_ concentrations. Three flasks as the blank were set to measure background GHG concentrations, correcting respired GHG from soils. Soil CO_2_, N_2_O, and CH_4_ concentrations were determined with a gas chromatograph, connected to an electron capture detector for N_2_O determination and a flame ionization detector for CH_4_ and CO_2_ determination. Certified gas standards within the range of the gas samples were used to calibrate the gas chromatograph system and minimize measurement errors.

The total GHG emission was estimated by assessing a global warming potential (GWP) for CH_4_ of 27 CO_2_ equivalents (CO_2_ eq) and for N_2_O of 273 CO_2_ eq (IPCC 2021).

### 2.4 Microbial PLFA Composition

To investigate the role of microbial composition in soil GHG production, the measurement of phospholipid fatty acid (PLFA) was conducted, since the PLFA method was more reliable for detecting rapid changes of microbial abundances from living communities (Siles et al. 2024). The protocol described in Frostegård, Tunlid, and Bååth (1993) and Nilsson et al. (2007). Briefly, 5.0 g freeze dried soil sample was extracted twice with 10 mL one‐phase Bligh and Dyer solution (CHCl_3_: MeOH: buffer, 1:2:0.8 v/v/v). The phospholipids were separated from the neutral lipid and glycolipids on a pre‐packed silica column using 1.5 mL trichloromethane, 6 mL acetone and 1.5 mL methanol, respectively. Then the fatty acids bonded to the phospholipids was separated from the backbone and transferred to methyl esters, to which methyl nonadecanoate fatty acid (19,0) as an internal standard was added. The derived fatty acid methyl esters (FAMEs) were finally dissolved in 0.3 mL *n*‐hexane and quantified on a Gas Chromatograph with flame ionization detector. PLFAs i14:0, 14:1ω5c, i15:0, a15:0, 15:1ω6c, i16:0, 16:1ω9c, 16:1ω7c, i17:0, a17:0, 17:1ω8, 17:0, 10Me17:0, 18:1ω7c, and 10Me18:0 were used to estimate bacterial abundance, whereas PLFAs 18:2ω6c and 18:1ω9c were used to estimate fungal abundance (Frostegård and Bååth 1996; Ruess and Chamberlain 2010). PLFA 16:1ω5c was represented to estimate arbuscular mycorrhizal (AM) fungi (Olsson 1999). PLFAs i14:0, i15:0, a15:0, i16:0, i17:0, and a17:0 were used to estimate gram‐positive bacteria, whereas 14:1ω5c, 15:1ω6c, 16:1ω9c, 16:1ω7c, 17:1ω8c, 18:1ω5c, and 18:1ω7c were used to estimated gram‐negative bacteria (Wilkinson et al. 2002). The estimation of actinomycetes was qualified by PLFAs 10Me17:0 and 10Me18:0 (Andersen and Petersen 2009). The lipid representative for anaerobic bacteria were assigned according to Vestal and White (1989) and Navarrete et al. (2000).

### 2.5 Enzyme Activity Measurements

Dehydrogenase as an intracellular enzyme indicated active microbial biomass, helping for the evaluation of oxidative metabolism associated with soil GHG production (Heitkötter et al. 2017). Dehydrogenase activity was assessed using the method described by Beyer et al. (1993), with the unit of μg TPF g^−1^ soil day^−1^. A sample of 5.0 g fresh soil was weighed into a container, to which 2 mL of 1% 2,3,5‐triphenyltetrazolium chloride (TTC) and 2 mL of 0.5 M TRIS buffer (pH 7.4) were administered, before a 24‐h incubation at 37°C without light. The triphenyl‐formazan produced from the reduction of TTC was extracted using 20 mL methanol, followed by shaking and filtering. Filtrates were measured at 485 nm absorbance using an ultraviolet spectrometer. Fluorescein diacetate (FDA) was thought to be hydrolyzed by various enzymes, and thereby FDA hydrolysis was widely accepted as an accurate approach for estimating total microbial activity (Wilkerson and Olapade 2020). FDA hydrolysis activity was determined by the optimized FDA hydrolysis method, expressed as μg FDA g^−1^ soil day^−1^ (Wilkerson and Olapade 2020). 5.0 g fresh soil was treated with 50 mL of 60 mM sodium phosphate buffer (pH 7.6) and 0.5 mL of 5 mM FDA substrate solution, followed by shaking on an incubator at 30°C for 24 h. Then 3 mL acetone was added to end FDA activity and the mixture was centrifuged at 10000 g for 5 min. The supernatant was measured at 490 nm absorbance using an ultraviolet spectrometer.

Sucrase was thought to be involved in soil C mineralization, playing a crucial role in CO_2_ release (Yang and Lu 2022). Sucrase activity was measured by a 3,5‐dinitrosalicylic acid colorimetric method, expressed as μg glucose g^−1^ soil day^−1^ (Guan 1986). 5.0 g fresh soil was weighted into a container, to which 15 mL glucose solution, 5 mL of 0.2 M sodium phosphate buffer (pH 5.5) and five drops of toluene were administered before an incubation at 37°C for 24 h. After incubation, the mixture was filtered and the filtrate was reacted with 3 mL 3,5‐dinitrosalicylate followed by heating for 5 min. The mixture was measured at 540 nm absorbance using an ultraviolet spectrometer. Urease as one of important N‐acquisitioning enzymes, was responsible for N cycling (Wang et al. 2020a). Soil urease activity was assessed by determining ammonium concentration released from soils based on the phenol blue colorimetric method (Zhou et al. 2022). A sample of 5.0 g fresh soil was weighed into a container, to which 5 mL of 1 M potassium citrate buffer (pH = 6.7) and 5 mL of 0.5 M urease solution were added, followed by an incubation at 37°C for 24 h in the dark. After incubation, the filtrate was treated with 4 mL of 1.35 M sodium phenol solution and 3 mL of 0.9% sodium hypochlorite solution. Ammonium concentration was measured at 578 nm absorbance using an ultraviolet spectrometer.

The catalase activity characterized redox ability of soils, related to microbial decomposition of SOM (Nowak et al. 2004). Soil catalase activity was determined using back titration residual H_2_O_2_ with 0.1 M potassium permanganate titration, expressed in mL KMnO_4_ g^−1^ soil day^−1^(Guan 1986). Phosphatase was P‐acquisitioning enzyme that targeted phosphate esters in SOM, which could result in synchronous mineralization of SOC due to the same source pools of organic P and C (Yang and Lu 2022). Soil acid and alkaline phosphatase activities were measured using 5.0 g fresh soil by the sodium phenyl phosphate colorimetry, expressed as μg phenol g^−1^ soil day^−1^ (Guan 1986). The soil sample was treated with 2.5 mL toluene and 20 mL of 0.5% buffered disodium phenyl phosphate (pH 5.4 for acid phosphatase; pH 8.0 for alkaline phosphatase). The mixture was incubated at 37°C for 24 h before added 100 mL of 0.3% aluminum sulfate solution. The filtrate was measured at 660 nm absorbance using an ultraviolet spectrometer.

### 2.6 Data Analysis

The effects of vegetation type and soil depth on soil characteristics, soil GHG emissions, *q*CO_2_, microbial PLFAs and soil enzyme activities were tested by two‐way analysis of variance (ANOVA). Before analysis, all the dependent variables were first log‐transformed to meet assumptions of normality and homogeneity of variance. Treatment comparisons of significant effects were conducted using Tukey's HSD pairwise comparisons at the *α* = 0.05 level. The relationship between soil GHG production and soil characteristics was estimated using a Pearson correlation analysis (*p* = 0.05). Data processing was conducted using Microsoft Excel 2019 and SPSS 18.0 (IBM, Chicago, USA). Data graphing was performed using Origin version 9.1 (OriginLab Corporation, Northampton, USA).

## 3 Results

### 3.1 Soil Characteristics

Compared to natural grassland, the SWC was reduced for artificial vegetation (*vegetation type p* < 0.01), with a more pronounced reduction by ca. 30% at 0–20 cm for *Prunus mume* (Table 1). Artificial vegetation had lower SOC content than that for natural grassland (*vegetation type p* < 0.001), with the smallest SOC for *Medicago sativa* (Table 1). Natural grassland and *Prunus mume* had the highest MBC and MBC/SOC, whereas *Prunus mume* had the highest DOC and DOC/SOC (*vegetation type* all *p* < 0.001, Table 1). Natural grassland and *Prunus mume* had higher TDN content ranged from ca. 8 μg Ng^−1^ soil to ca. 20 μg Ng^−1^ soil, while *Medicago sativa* had lower TDN ranged from ca. 3 μg Ng^−1^ soil to ca. 20 μg Ng^−1^ soil (*vegetation type p* < 0.001, Table 1). At 0–30 cm, *Armeniaca sibirica* had more than two‐times higher DOC/TDN than that for natural grassland. DOC/TDN increased with soil depth (*p* < 0.001), with the most pronounced increment for *Medicago sativa* , resulting in the highest ratio of ca. 58 at 30–40 cm (Table 1). The contents of SOC, MBC, DOC and TDN declined with soil depth for all the types of vegetation (all *p* < 0.001).

**TABLE 1:** Soil characteristics at different depths following five types of vegetation restoration on the Loess Plateau.

Soil depth (cm)	Vegetation type	SWC (g H_2_O g^−1^ soil)	SOC (mg C g^−1^ soil)	MBC (μg C g^−1^ soil)	DOC (μg C g^−1^ soil)	TDN (μg Ng^−1^ soil)	MBC/SOC	DOC/SOC	DOC/TDN
0–10	Natural grassland	0.23 ± 0.01a	16.6 ± 1.40a	664 ± 50.6a	196 ± 19.0ab	22.0 ± 3.66a	0.048 ± 0.002a	0.012 ± 0.001c	9.8 ± 2.66ab
	*Armeniaca sibirica*	0.21 ± 0.02ab	13.8 ± 1.51ab	300 ± 15.5b	186 ± 1.0ab	18.5 ± 5.91a	0.022 ± 0.001b	0.014 ± 0.001bc	17.7 ± 5.75a
	*Prunus mume*	0.16 ± 0.00c	11.4 ± 0.83bc	584 ± 13.6a	230 ± 3.9a	18.1 ± 1.62a	0.052 ± 0.003a	0.020 ± 0.001a	13.0 ± 1.43ab
	*Caragana korshinskii*	0.18 ± 0.00bc	10.8 ± 0.81bc	278 ± 20.6b	204 ± 7.7ab	20.0 ± 5.25a	0.026 ± 0.003b	0.017 ± 0.000abc	12.8 ± 4.93ab
	*Medicago sativa*	0.19 ± 0.00bc	8.2 ± 1.41c	222 ± 20.4b	161 ± 11.5b	24.9 ± 1.78a	0.028 ± 0.005b	0.018 ± 0.002ab	6.5 ± 0.56b
10–20	Natural grassland	0.21 ± 0.01a	13.1 ± 0.11a	372 ± 40.0b	183 ± 5.7b	16.4 ± 3.83a	0.031 ± 0.001b	0.014 ± 0.000c	12.8 ± 3.48b
	*Armeniaca sibirica*	0.18 ± 0.01ab	10.7 ± 0.42b	151 ± 28.8c	176 ± 7.2b	4.7 ± 0.67b	0.014 ± 0.003c	0.017 ± 0.001bc	38.7 ± 5.69a
	*Prunus mume*	0.14 ± 0.02b	8.6 ± 0.77bc	483 ± 19.1a	335 ± 20.8a	19.4 ± 3.01a	0.057 ± 0.007a	0.043 ± 0.003a	15.9 ± 2.56b
	*Caragana korshinskii*	0.17 ± 0.01ab	8.6 ± 0.53bc	171 ± 14.4c	169 ± 5.0b	7.0 ± 1.47b	0.020 ± 0.001bc	0.020 ± 0.001bc	27.5 ± 7.81ab
	*Medicago sativa*	0.15 ± 0.01b	6.9 ± 0.14c	201 ± 15.9c	161 ± 7.6b	4.7 ± 0.42b	0.028 ± 0.001bc	0.023 ± 0.001b	34.9 ± 3.12a
20–30	Natural grassland	0.18 ± 0.01a	12.3 ± 0.18a	294 ± 28.9b	160 ± 15.4b	10.1 ± 1.56a	0.028 ± 0.006b	0.013 ± 0.001d	16.9 ± 3.73b
	*Armeniaca sibirica*	0.17 ± 0.01a	10.6 ± 0.30b	116 ± 16.4c	161 ± 9.2b	4.3 ± 0.95b	0.011 ± 0.002c	0.015 ± 0.001 cd	41.8 ± 9.36a
	*Prunus mume*	0.13 ± 0.03a	6.7 ± 0.33 cd	410 ± 26.8a	223 ± 20.5a	8.0 ± 1.50ab	0.061 ± 0.002a	0.033 ± 0.002a	29.5 ± 5.07ab
	*Caragana korshinskii*	0.17 ± 0.01a	7.1 ± 0.47c	122 ± 13.1c	156 ± 14.3b	6.1 ± 1.58ab	0.017 ± 0.002bc	0.022 ± 0.002bc	28.8 ± 6.28ab
	*Medicago sativa*	0.16 ± 0.01a	5.4 ± 0.24d	168 ± 4.2c	159 ± 6.5b	3.6 ± 0.78b	0.031 ± 0.002b	0.029 ± 0.002ab	47.8 ± 9.97a
30–40	Natural grassland	0.16 ± 0.01a	9.7 ± 1.16a	278 ± 52.2a	161 ± 9.8b	5.4 ± 0.92ab	0.036 ± 0.004a	0.017 ± 0.001b	32.6 ± 8.72a
	*Armeniaca sibirica*	0.16 ± 0.00a	10.2 ± 0.08a	86 ± 5.5b	160 ± 1.9b	5.2 ± 1.78ab	0.008 ± 0.001c	0.016 ± 0.000b	39.7 ± 14.10a
	*Prunus mume*	0.17 ± 0.00a	6.7 ± 0.81ab	171 ± 30.1ab	201 ± 2.2a	8.0 ± 0.04a	0.027 ± 0.002ab	0.030 ± 0.000ab	25.2 ± 0.38a
	*Caragana korshinskii*	0.15 ± 0.02a	7.1 ± 0.94ab	77 ± 17.2b	151 ± 10.4b	4.8 ± 0.83ab	0.011 ± 0.002c	0.025 ± 0.007ab	33.8 ± 6.63a
	*Medicago sativa*	0.13 ± 0.01a	4.8 ± 0.69b	107 ± 4.1b	154 ± 5.3b	2.9 ± 0.58b	0.023 ± 0.003b	0.033 ± 0.004a	58.3 ± 12.40a

*Note:* Lowercase letters indicate significant differences between treatments for each soil depth based on Tukey's HSD pairwise comparisons.

### 3.2 Soil Greenhouse Gas Emissions

**FIGURE 1:**
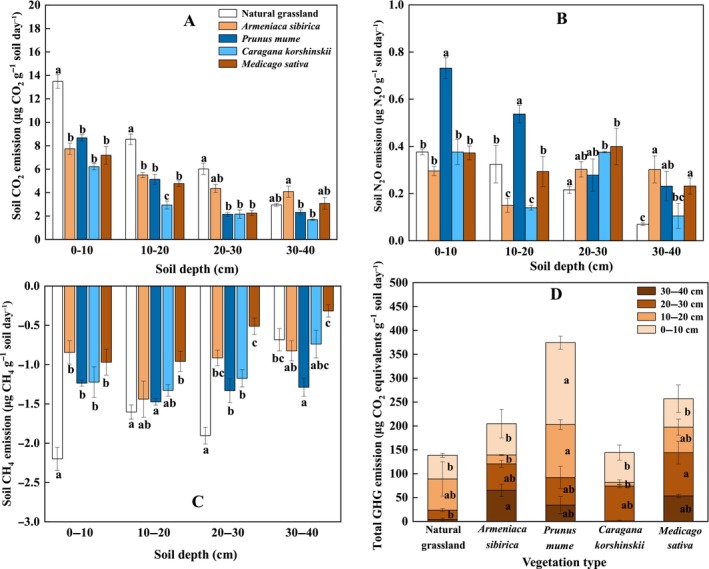
Emissions of soil CO_2_ (panel A), N_2_O (panel B), CH_4_ (panel C), and total greenhouse gas (panel D) at different depths following five types of vegetation restoration on the Loess Plateau. For CH_4_, negative values indicate the consumption by soils. Lowercase letters indicate significant differences between treatments for each soil depth based on Tukey's HSD pairwise comparisons.

The emissions of soil CO_2_, N_2_O, CH_4_, and total GHG were all affected by vegetation type, soil depth and their interactions (all *p* < 0.05, Figure 1). CO_2_ emissions decreased with soil depth. Artificial vegetation had lower CO_2_ emissions than that for natural grassland, with a significant reduction at 0–30 cm for *Caragana korshinskii* (Figure 1A). *Prunus mume* had higher soil N_2_O than other types of vegetation, which was more pronounced at 0–20 cm with the level ranged from 0.5 to 0.7 μg g^−1^ soil day^−1^ (Figure 1B). *Armeniaca sibirica* and *Caragana korshinskii* had the smallest soil N_2_O ranged from 0.1 to 0.3 μg g^−1^ soil day^−1^ at 0–20 cm, while natural grassland had the lowest soil N_2_O ranged from 0.1 to 0.2 μg g^−1^ soil day^−1^at 20–40 cm.

Soil CH_4_ emissions were negative for all the vegetation types, showing a CH_4_ consumption (Figure 1C). At 0–30 cm, natural grassland had the highest CH_4_ consumption (ca. 2 μg g^−1^ soil day^−1^), compared to the lowest consumption of ca. 1 μg g^−1^ soil day^−1^ for *Medicago sativa* . At 30–40 cm, soil CH_4_ consumption decreased compared to the top layer except the case of *Prunus mume* , resulting in the highest consumption of ca. 1.5 μg g^−1^ soil day^−1^ for *Prunus mume* . The responses of total soil GHG were positive across all the types of vegetation, where *Prunus mume* had higher total GHG emissions that were mainly derived from the layer of 0–20 cm, while *Caragana korshinskii* and natural grassland had smaller total GHG emissions (Figure 1D).

### 3.3 Microbial Community Composition

**FIGURE 2:**
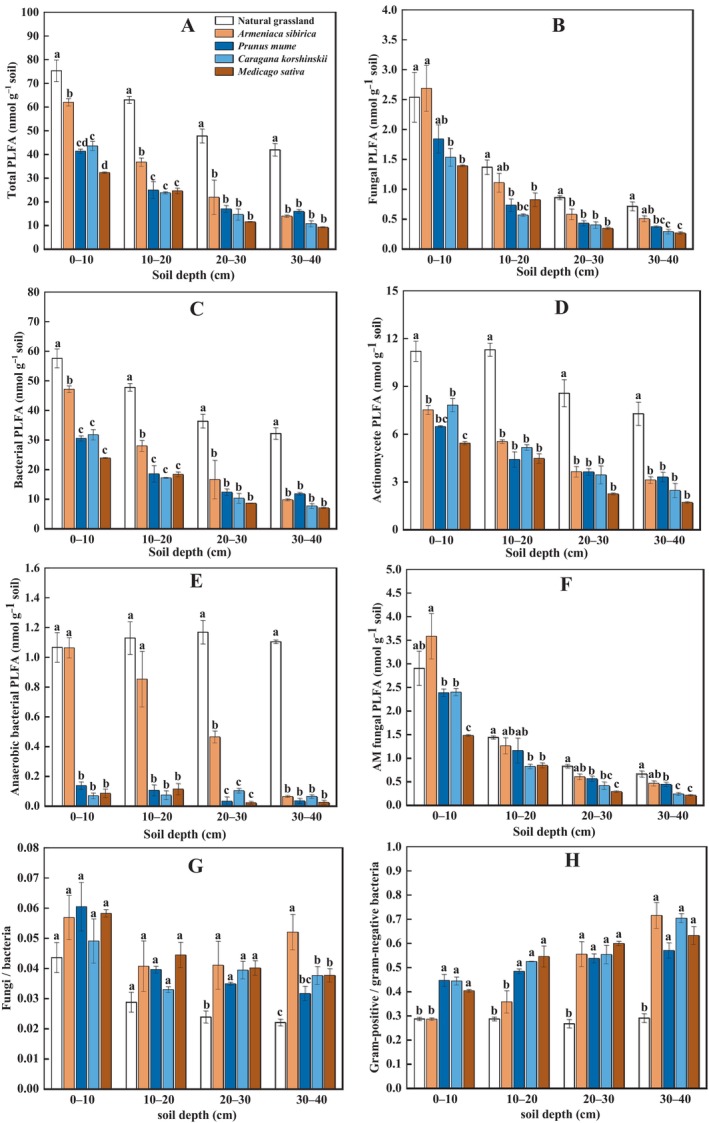
The relative abundance of soil microbial PLFAs including total PLFAs (panel A), fungal PLFAs (panel B), bacterial PLFAs (panel C), actinomycete PLFAs (panel D), anaerobic bacterial PLFAs (panel E), arbuscular mycorrhizal fungal PLFAs (AM fungi, panel F), the ratio of fungal to bacterial PLFAs (panel G), and the ratio of gram‐positive to gram‐negative bacterial PLFAs (panel H) at different depths following five types of vegetation restoration on the Loess Plateau. Lowercase letters indicate significant differences between treatments for each soil depth based on Tukey^'^s HSD pairwise comparisons.

Microbial PLFAs were affected by vegetation type (all *p* < 0.001) and decreased with soil depth (all *p* < 0.001), where natural grassland had the highest total PLFAs and PLFAs of fungi, bacteria and AM fungi (Figure 2A–F; Figure S1). In particular, the PLFAs of actinomycetes and AM fungi for natural grassland were both more than 2‐times higher than *Medicago sativa* throughout the soil profile (0–40 cm) (Figure 2D,F). Natural grassland had ca. 10‐times higher anaerobic bacterial PLFAs at 0–40 cm, compared to that for *Prunus mume* , *Caragana korshinskii*, and *Medicago sativa* (Figure 2E). Among the artificial vegetation, total PLFAs and bacterial PLFAs at 0–20 cm for *Armeniaca sibirica* were both over 100% higher than that for *Medicago sativa* (Figure 2A,C). The PLFAs of fungi and AM fungi at 0–10 cm for *Armeniaca sibirica* were ca. 100% higher than that for *Medicago sativa* (Figure 2B,F).

Fungi/bacteria ratio varied with vegetation type and soil depth (both *p* < 0.001, Figure 2G). Artificial vegetation increased fungi/bacteria ratio compared to natural grassland, where *Armeniaca sibirica* and *Prunus mume* both had ca. 40% higher ratio at 0–20 cm, while *Armeniaca sibirica* had over 70% higher ratio at 20–40 cm. Gram‐positive/gram‐negative bacteria ratio was affected by vegetation type and soil depth (both *p* < 0.001), as well as their interactions (*p* < 0.01, Figure 2H). At 0–20 cm, gram‐positive/g‐negative bacteria ratio was increased by over 35% for all of *Prunus mume* , *Caragana korshinskii*, and *Medicago sativa* , compared to natural grassland and *Armeniaca sibirica*. At 20–40 cm, all the artificial vegetation had more than two‐times higher gram‐positive/g‐negative bacteria ratio than that for natural grassland.

### 3.4 Soil Enzyme Activity

**FIGURE 3:**
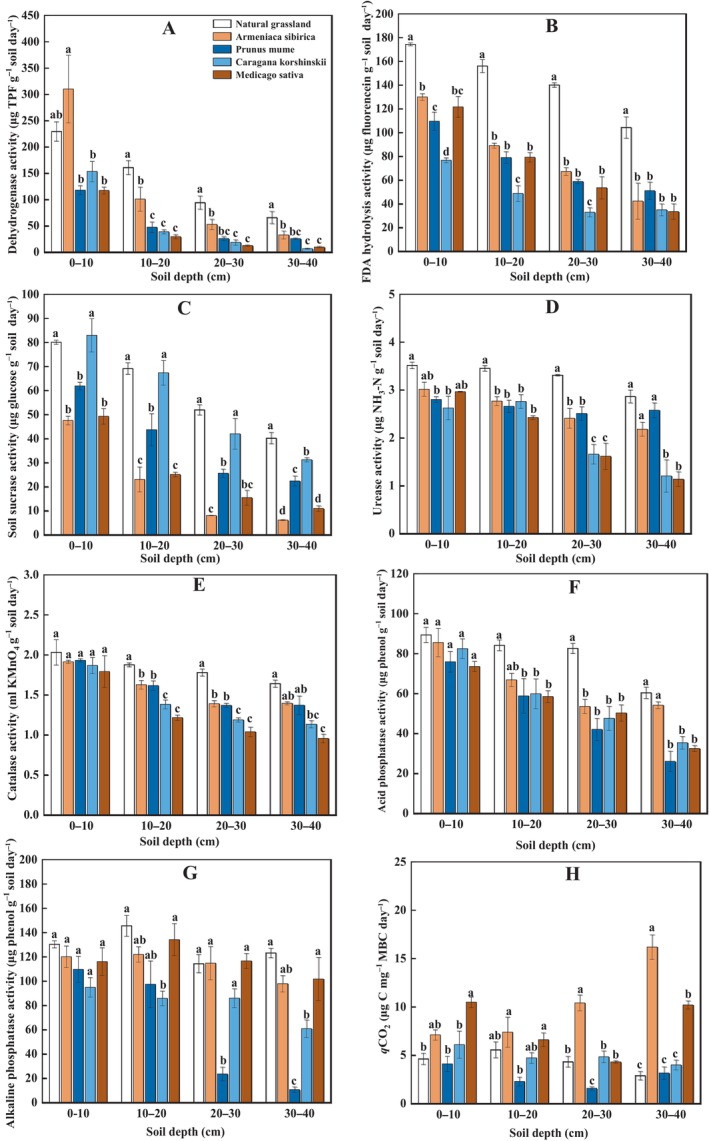
Soil enzyme activity (panel A–G) and *q*CO_2_ (panel H) at different depths following five types of vegetation restoration on the Loess Plateau. Lowercase letters indicate significant differences between treatments for each soil depth based on Tukey's HSD pairwise comparisons.

Enzyme activities were affected by vegetation type (all *p* < 0.001), and decreased with soil depth (all *p* < 0.001, Figure 3). In general, natural grassland had higher enzyme activities compared to artificial vegetation. In natural grassland, dehydrogenase activity was ca. 2‐times higher at 0–10 cm and ca. 10‐times higher at 30–40 cm, respectively, compared to the lowest level for both of *Caragana korshinskii* and *Medicago sativa* (Figure 3A). *Caragana korshinskii* had the smallest FDA hydrolysis activity ranged from ca. 30 to 80 μg g^−1^ soil day^−1^, compared to the highest activity ranged from 100 to 170 μg g^−1^ soil day^−1^ for natural grassland (Figure 3B). Natural grassland and *Caragana korshinskii* both had higher sucrase activity than other types of vegetation (Figure 3C). *Caragana korshinskii* and *Medicago sativa* had lowest urease and catalase activities, with the most pronounced reduction of urease by ca. 50% at 20–40 cm (Figure 3D), and most pronounced reduction of catalase by ca. 40% at 10–40 cm, respectively, compared to natural grassland (Figure 3E). The differences of acid phosphatase activity between artificial vegetation types were not significant (Figure 3F). *Prunus mume* had the lowest activity of alkaline phosphatase, with the especial case for the depth of 20–40 cm (Figure 3G).

A*rmeniaca sibirica* and *Medicago sativa* had the highest *q*CO_2_, while *Prunus mume* had the lowest *q*CO_2_ (*vegetation type p* < 0.01, Figure 3H). Specifically, *q*CO_2_ for *Armeniaca sibirica* and *Medicago sativa* reached the greatest level at 30–40 cm, which was ca. 5‐times higher and ca. 3‐times higher than other types of vegetation, respectively (Figure 3H).

### 3.5 Key Soil Factors Influencing GHG Emissions

Soil CO_2_ emissions and CH_4_ consumption both had positive and highly significant correlations with SWC, SOC, MBC and TDN (all *p* < 0.01) as well as MBC/SOC (*p* < 0.05), and had negative correlations with DOC/SOC and DOC/TDN (both *p* < 0.01, Table 2). CH_4_ consumption was also positively correlated with DOC (*p* < 0.05). N_2_O emissions had positive and highly significant correlations with MBC, DOC, TDN and MBC/SOC (all *p* < 0.01), and had a negative correlation with DOC/TDN (*p* < 0.05, Table 2). Total GHG emissions were positively correlated with MBC, DOC, TDN, and MBC/SOC (all *p* < 0.01, Table 2).

**TABLE 2:** Pearson product–moment correlation coefficients between soil characteristics and greenhouse gas after vegetation restoration.

GHG	SWC	SOC	MBC	DOC	TDN	MBC/SOC	DOC/SOC	DOC/TDN
CO_2_	0.542**	0.757**	0.591**	0.152	0.565**	0.250*	−0.437**	−0.434**
CH_4_	0.398**	0.551**	0.580**	0.266*	0.394**	0.306*	−0.334**	−0.459**
N_2_O	0.085	0.180	0.505**	0.392**	0.411**	0.453**	0.090	−0.247*
Total GHG	0.007	0.189	0.444**	0.420**	0.367*	0.409**	0.135	−0.156

* Significant correlation (α = 0.05).

** Highly significant correlation (α = 0.01).

**FIGURE 4:**
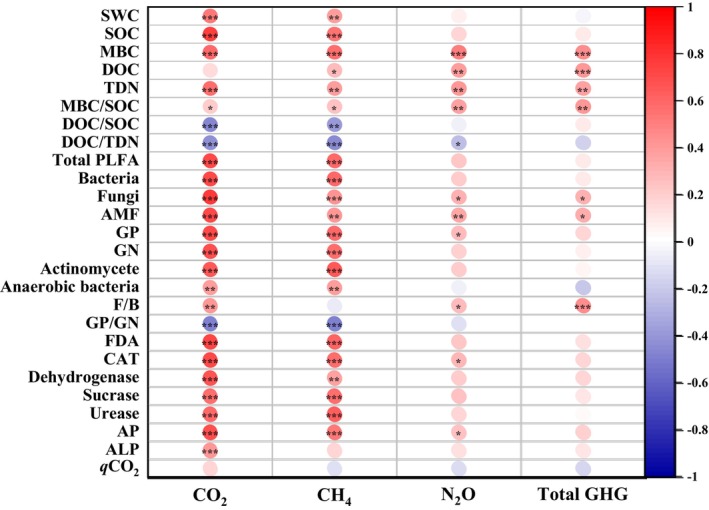
Pearson correlation analysis between greenhouse gas and properties of soil biochemistry and microbial composition after vegetation restoration (GP, gram‐positive bacteria; GN, gram‐negative bacteria; F/B, fungi/bacteria, AMF, arbuscular mycorrhizal fungi; CAT, catalase; AP, acid phosphatase; ALP, alkaline phosphatase). The intensity of color indicates the correlation coefficient (blue and red indicate negative and positive correlation, respectively. G). *, ** and *** indicates *p* < 0.05, 0.01 and 0.001, respectively.

In general, soil CO_2_ emissions were significantly and positively correlated with microbial communities and enzyme activities, where the correlation coefficient of CO_2_ was lower with anaerobic bacteria and fungi/bacteria (both *p* < 0.01) than other factors (all *p* < 0.001, Figure 4). Soil CH_4_ consumption was also correlated with microbial communities and enzyme activities, where the correlation coefficient of CH_4_ was lower with AM fungi, anaerobic bacteria and dehydrogenase (all *p* < 0.01) than other factors (all *p* < 0.001, Figure 4). In addition, soil CO_2_ emission and CH_4_ consumption both had a negative correlation with gram‐positive/g‐negative bacteria (*p* < 0.001, Figure 4). Soil N_2_O emissions were positively and significantly correlated with fungi, gram‐positive bacteria, fungi/bacteria, catalase and acid phosphatase (all *p* < 0.05), as well as AM fungi (*p* < 0.01). Total GHG emissions had positive correlations with fungi and AM fungi (both *p* < 0.05), and fungi/bacteria (*p* < 0.001, Figure 4).

## 4 Discussion

### 4.1 Effects of Vegetation Restoration on Soil CO_2_ Emissions

Soil CO_2_ emissions were higher for natural grassland than artificial vegetation, which was more pronounced at 0–30 cm (Figure 1A). This finding seemingly contradicted some studies where natural restoration had higher potentials for mitigating CO_2_ release in arid and semiarid regions (Zhang et al. 2022; Zhou et al. 2023), but similar results were also reported in previous researches (Han and Zhu 2020; Feng et al. 2022). This response could be on one hand associated with positive dependence of microbial‐controlled decomposition on moisture (Figure 4) (Schimel 2018; Na et al. 2021). In our study, SWC was higher for natural grassland than that for artificial vegetation, indicating that soil moisture could be a controller of microbial decomposition of SOC (Table 1). This was likely because natural grassland had lower root biomass and thus soil water consumption, compared to managed plantations and leguminous pasture of *Medicago sativa* , maintaining water sources and active microbial decomposition (Brümmer et al. 2012; Huang et al. 2019). On the other hand, vegetation types might affect SOC mineralization driven by differences in plant inputs, generating variations in SOC quality that associated with the amount of decomposable C (Kuzyakov 2002; Na et al. 2022). Higher quality of SOC had been related to more pronounced SOC mineralization (Na et al. 2022). We found that natural grassland showed a higher MBC/SOC ratio than most of artificial vegetations (Table 1), coinciding with the pronounced CO_2_ emissions (Figure 4), indicating higher microbial assimilability that promoted SOC mineralization, since the MBC/SOC ratio had been proposed as a representative of SOC quality (Hobbie and Hobbie 2013). Although natural grassland had larger soil CO_2_ emissions, but generated higher soil C contents (Table 1). These findings suggested that the restoration of natural grassland possibly acted as a double‐edge sword for soil C pool, where natural vegetation restoration could drive soil C accrual, but also cause C loss.

We also found that natural grassland had higher total microbial PLFAs (Figure 2A) and enzyme activities (Figure 3), matching the stronger soil CO_2_ emissions. This result indicated that natural vegetation restoration might largely enhance microbial growth and metabolism, resulting in an improvement in SOC decomposition. In addition, natural grassland showed a greater bacterial abundance and a lower fungi/bacteria ratio, compared to artificial vegetation (Figure 2G), highlighting a shift in soil microbial composition induced by different modes of vegetation restoration. After natural restoration, bacteria likely became a dominant agent responsible for SOC mineralization, consistent with the study where bacteria were found to play a more active role in soil C turnover following vegetation restoration in arid regions (Yu et al. 2023). Among the bacterial phyla, natural grassland had the most pronounced abundance of actinomycetes (Figure 2D) and the lowest gram‐positive/g‐negative bacteria ratio (Figure 2H). These results implied that actinomycetes and gram‐negative bacteria might be specifically involved in SOC decomposition. Gram‐negative bacteria had been found to grow fast and rely more on readily degradable plant C sources that were more abundant in grassland rather than woodland, thus contributing to plant‐derived SOM mineralization (Kramer and Gleixner 2008). By comparison, actinomycetes turned over slowly and preferred to decompose recalcitrant organic compounds (Bhatti et al. 2017). Studies found that actinomycetes could feed on gram‐negative bacterial necromass via food web, increasing their growth and activities (Kindler et al. 2006; Zheng et al. 2021). As such, elevated CO_2_ in natural grassland could be partly resulted from the decomposition of gram‐negative bacterial residue‐derived components in SOM modulated by actinomycetes.

In contrast with the natural grassland, leguminous *Caragana korshinskii* had the smallest soil CO_2_ emissions across five types of vegetations (Figure 1A), consistent with studies where deep‐rooted legume trees could foster soil C sequestration (Chai et al. 2019; Kong et al. 2022). This was likely associated with the N‐fixing ability of legume plants, which might alleviate microbial N limitation resulted from the competition for resources between roots and microorganisms after vegetation restoration, thereby lowering microbial demands for N from SOM and thus SOC mineralization (Na et al. 2022; Gou et al. 2024). This was further evidenced by lower N‐acquisitioning enzyme activity such as urease, compared to natural grassland (Figure 3D). Moreover, compared to the natural grassland, plantations of *Caragana korshinskii* had lower AM fungal PLFAs (Figure 2F). Previous studies reported that AM fungi tended to decline with N deposition (Pan et al. 2020; Andrade‐Linares et al. 2023). Thus, there could be a small stimulation on AM fungal growth in a less N‐limited condition from legume systems (Gou et al. 2024). In addition, the cultivated practices in managed plantations probably resulted in hyphal disruption, while the high plant diversity in natural grassland might generate great AMF colonization contributing to SOC decomposition (Carrillo et al. 2016; Hu et al. 2020). Together, these findings indicated that artificial restoration was more conducive to mitigating CO_2_ emissions than natural restoration in degraded ecosystems, of which legume shrubland had greatest potentials for soil C sequestration.

### 4.2 Effects of Vegetation Restoration on Soil N_2_O Emissions

The plantation of *Prunus mume* had higher soil N_2_O emissions, whereas natural grassland had relatively lower N_2_O emissions (Figure 1B), suggesting that the restoration of natural grassland could be a more appropriate selection for N_2_O emission cut in the arid and semiarid regions. The production of soil N_2_O was commonly limited by N availability including NH_4_^+^ and NO_3_^−^ that were precursors of nitrification and denitrification, respectively (Shcherbak and Robertson 2019). In our study, *Prunus mume* had high TDN content consisting largely of mineral N (Table 1), which might exaggerate N_2_O evolution (Chen et al. 2018). Meanwhile, compared to the natural grassland, *Prunus mume* had a higher DOC/SOC ratio (Table 1) but lower total microbial PLFA (Figure 2). These responses suggested that there could be a large number of available resources provided for a small size of living microbial community after plantations of *Prunus mume* , thereby satisfying resource demands for nitrifiers or denitrifiers and stimulating their activities (Jäger et al. 2011; Shcherbak and Robertson 2019).

Our results revealed that *Prunus mume* had higher fungi/bacteria ratio at 0–30 cm than natural grassland (Figure 2G), coinciding with the pronounced soil N_2_O emissions. These findings suggested that N_2_O emissions might be triggered via fungal pathway after vegetation restoration. Similar findings were also reported in studies where fungal denitrification for N_2_O production was identified to be dominant in semiarid soils (McLain and Martens 2006; Hayatsu et al. 2008). In the plantation of *Prunus mume* , soil moisture was lower than other types of vegetation (Table 1). Under this condition, fungi could be more competitive than bacteria in N processing, due to their greater metabolic capacity under low water potentials (Hayatsu et al. 2008). In addition, a meta‐analysis study reported that vegetation restoration on the Loess Plateau decreased soil pH compared to undisturbed soils (Sha et al. 2023), which might favor fungal growth considering its preference for acidic environments (Rousk et al. 2009). The high soil N_2_O emissions from *Prunus mume* plantations could be also associated with an increase in gram‐positive/gram‐negative bacteria ratio, compared to natural grassland (Figure 2H). This finding pointed out the key contribution of gram‐positive bacteria to N_2_O evolution, as studies confirmed that gram‐positive bacteria contain denitrifying members such as *Bacillu* (Verbaendert et al. 2014; Mania et al. 2014). These changes in microbial composition could be attributed to the differences in microbial substrate preference between woodland and grassland (Bai et al. 2024). The substrates derived from plant litter with high lignocellulose in woody *Prunus mume* might be preferentially selected by fungi and gram‐positive bacteria (Faust et al. 2018), since fungi and gram‐positive bacteria had been found to be capable of breaking down complex plant biopolymers (Kramer and Gleixner 2008). Furthermore, in *Prunus mume* plantations, alkaline phosphatase activity was lower than natural grassland, indicating a higher P limitation in undisturbed soils (Figure 3G). Differently, previous studies reported that afforestation on the Loess Plateau resulted in soil P limitation, enhancing the mineralization of soil calcium phosphate by stimulating alkaline phosphatase (Xu et al. 2022; Zhang et al. 2024). Our results therefore indicated that the changes in microbial nutrient metabolism in soils could vary depending on restoration species. Together, soil NO_2_ emissions could be strengthened after artificial vegetation restoration such as plantations of *Prunus mume* , posing a threat to global warming.

### 4.3 Effects of Vegetation Restoration on Soil CH_4_ Emissions

Soil CH_4_ emissions were negative throughout the soil profile across different types of vegetations (Figure 1C), indicating soil consumption of CH_4_ acted as a sink after vegetation restoration. Soil uptake of CH_4_ mainly depended on CH_4_‐oxidizing process, given that most of CH_4_ produced from soils were consumed as energy sources by methanotrophs before it migrated to the atmosphere (Le Mer and Roger 2001; Malyan et al. 2016). This negative CH_4_ emission might be therefore resulted from enhanced CH_4_ oxidation by prolonged drought on the Loess Plateau, because diffusion of CH_4_ and oxygen could be increased through improved porosity by low water contents (Borken et al. 2006; Megonigal and Guenther 2008). Notably, grassland had higher soil CH_4_ consumption, compared to artificial vegetation, particularly for *Medicago sativa* with the smallest CH_4_ consumption (Figure 1C). These contrasting findings indicated that there could be stronger oxidation governing CH_4_ flux after natural restoration, consistent with studies where CH_4_ oxidation rate was greater in undisturbed soils rather than disturbed soils (Tate 2015; Feng et al. 2022). This might be because the disturbance like revegetation in ecosystems led to a decrease in methanotroph diversity, lowering soil CH_4_ oxidation rates (Tate 2015). During biological oxidation, CH_4_ was able to be converted into CO_2_ released from soils (Kallistova et al. 2017). As such, the large CO_2_ emissions after natural grassland restoration might further confirm the great CH_4_ oxidation. These distinct effects of vegetation restoration on CH_4_ evolution could be explained by changes in soil moisture associated with the legacy of drought and root‐water uptake (Feng et al. 2017; Bian et al. 2019). In dry climate regions, the wide root distribution in a system would reduce CH_4_ oxidation efficiency, due to water shortage caused by excess root‐water uptake that suppressed methanotroph activities (Bian et al. 2019). Thus, the relatively high soil moisture in natural grassland with narrow root distribution was likely a contributing factor to CH_4_ oxidation. In addition, the changes in soil structure after vegetation restoration could also affect soil CH_4_ uptake (Stiehl‐Braun et al. 2011; Karbin et al. 2017). Stiehl‐Braun et al. (2011) indicated that methanotroph preferred to assimilate CH_4_ on the surface of soil aggregates. It was discovered that artificial vegetation increased the fraction of macro‐aggregates in degraded soils compared to natural restoration, with a pronounced effect for *Medicago sativa* (Kan et al. 2023). Considering that macro‐aggregates were thought to have a smaller surface to volume ratio than micro‐aggregates (Karbin et al. 2017), the improved soil macro‐aggregates from *Medicago sativa* pasture might lead to less CH_4_ uptake.

In addition, the pronounced CH_4_ consumption in natural grassland could be associated with lower gram‐positive/gram‐negative bacteria ratio (Figure 4H). This finding indicated that active gram‐negative bacteria was seemingly responsible for CH_4_ oxidation after vegetation restoration, since gram‐negative bacteria involve methanotrophic populations such as *Alphaproteobacteria* and *Gammaproteobacteria* (Bodelier et al. 2000; Malyan et al. 2016). The activities of methane‐oxidizers were expected to be stimulated by high soil N availability (Bodelier et al. 2000; Xu et al. 2023). In our study, *Medicago sativa* had lower TDN contents at 10–40 cm compared to natural grassland (Table 1), suggesting that the lower N availability might inhibit methane‐oxidizing bacteria and thus CH_4_ consumption. Furthermore, there was higher anaerobic bacterial abundance in natural grassland than artificial vegetation, matching greater CH_4_ uptake in soils (Figures 2E and 4), which implied that CH_4_ oxidation might be linked to methanogenesis involved in anaerobic microflora. The uptake of CH_4_ by arid soils could be the consequence of methanotrophs utilizing CH_4_ as substrates for growth and activities (Wen et al. 2024). The high CH_4_ production might have induced a rapid proliferation of methanotrophic cell, resulting in an immediate increase in soil CH_4_ oxidation (Cai et al. 2016; Wen et al. 2024). In natural grassland, more anaerobic bacteria could thus drive methanogenesis process and provide large sources of CH_4_ for methanotrophs, enhancing CH_4_‐oxidizing efficiency. These findings suggested that vegetation restoration of natural grassland was favorable for CH_4_ mitigation, while artificial vegetations, especially the pasture of *Medicago sativa* , might lower the ability of soil CH_4_ uptake as a sink.

### 4.4 Effects of Vegetation Restoration on Total Soil GHG Emissions

Compared to artificial vegetation restoration, natural restoration of grassland resulted in an increase in CO_2_ emissions, but it decreased N_2_O and CH_4_ production. These findings indicated that different types of soil GHG responded differently when vegetation restoration was implemented. As such, assessing only one or two of soil GHG emissions cannot fully capture the impact of ecological restoration on soil GHG emissions and their contribution to climate change. The various GHG emissions ultimately led to positive total soil GHG responses (Figure 1D), suggesting a GHG source after vegetation restoration on the Loess Plateau. Partly consistent with our hypothesis, natural grassland and managed shrubland of *Caragana korshinskii* both had the lowest total GHG emissions, whereas artificial plantation of *Prunus mume* had the highest total GHG emissions. These results demonstrated that the restoration of artificial vegetation had potentials to reduce total GHG emissions as effectively as natural restoration, which was highly dependent on the type of vegetation species used. In addition, the pattern of total GHG emissions coincided with the dynamics of soil N_2_O emissions (Figure 1B), reflecting that the production of N_2_O might determine the GHG balance in soils after vegetation restoration to some extent. Thus, it could be effective to mitigate GHG emissions by the measures inhibiting N_2_O evolution during vegetation restoration. Taken together, the restoration of natural grassland and artificial N‐fixing shrubland might be recommended for GHG emission reduction in arid or semiarid regions, contributing to soil C sequestration and global warming mitigation.

### 4.5 Limitations and Future Research

The various responses of soil GHG emissions to vegetation restoration had an important effect on climate change and ecological sustainability. The current study might provide a limited insight into soil GHG dynamics, since it was observed at once without the sustained assessment. This could lead to challenges in predicting the resilience of the restored ecosystems to future disturbances and their feedback to climate change. Thus, a long‐term quantification of soil GHG production and soil properties should be performed in the future study. Additionally, the measurements of abiotic factors such as soil texture and mineralogy that influenced soil GHG emissions were ignored. To enhance the understanding of potential mechanisms by which vegetation restoration modulated soil GHG emissions, further work should consider the interactions of abiotic factors with microbes and vegetations as well was their effects on GHG production. Moreover, there was a lack of comparisons between restored and degraded lands in terms of GHG emissions and soil biochemical properties, which could affect the evaluation of outcomes from vegetation restoration efforts. As such, further investigation is necessary to examine the impact of vegetation restoration on soil GHG emissions.

## 5 Conclusions

The restoration of artificial vegetation increased emissions of soil N_2_O and CH_4_ (i.e., decrease in CH_4_ consumption), but decreased CO_2_ emissions, compared to the natural restoration of grassland on the Loess Plateau. These different responses of GHG emissions were largely associated with the changes in soil moisture, microbial composition and soil resource availability for microorganisms following vegetation restoration. In particular, the pronounced soil CO_2_ emissions in natural grassland could be attributed to higher MBC/SOC ratio that provided more decomposable C sources for dominated bacterial group such as gram‐negative bacteria and actinomycetes. In contrast, small soil CO_2_ production in plantations of leguminous *Caragana korshinskii* was likely linked to its great N‐fixing ability, which alleviated microbial demands for N from SOM. In addition, *Prunus mume* had high soil N_2_O emissions, mediated by active fungi and gram‐positive bacteria which was affected by N availability and DOC/SOC ratio. *Medicago sativa* had lower soil CH_4_ consumption, associated with lower N availability that might inhibit methane‐oxidizing bacteria. The uptake of soil CH_4_ was possibly dominated by anaerobic bacteria and gram‐negative bacteria. These differential responses of soil CO_2_, N_2_O and CH_4_ emissions ultimately led to the lowest total GHG for natural grassland and artificial shrubland of leguminous *Caragana korshinskii* , but the largest total GHG for plantations of *Prunus mume* , suggesting that the restoration of *Caragana korshinskii* and natural grassland on the Loess Plateau was favorable for GHG mitigation. These findings revealed that natural restoration and artificial restoration via leguminous shrub should be given priority in arid and semiarid regions when ecological restoration strategy was implemented. Overall, our study provided an important example of building the ecological restoration roadmap and forecasting GHG emissions caused by vegetation restoration from a broad landscape of fragile systems, supporting climate mitigation policies.

## Author Contributions

**Jihai Zhou:** conceptualization (equal), data curation (equal), investigation (equal), methodology (equal), supervision (equal), writing – original draft (equal). **Daokun Liu:** data curation (supporting), formal analysis (equal), software (supporting). **Shangqi Xu:** formal analysis (equal), visualization (supporting). **Xiaoping Li:** data curation (supporting), validation (supporting). **Jiyong Zheng:** investigation (equal), methodology (supporting). **Fengpeng Han:** investigation (equal), methodology (supporting). **Shoubiao Zhou:** conceptualization (supporting), supervision (equal). **Meng Na:** conceptualization (equal), data curation (equal), investigation (equal), methodology (equal), software (equal), supervision (equal), writing – original draft (equal), writing – review and editing (equal).

## Conflicts of Interest

The authors declare no conflicts of interest.

## Supporting information


**Figure S1.** Soil gram‐positive and gram‐negative bacterial PLFA abundance at different depths following five types of vegetation restoration on the Loess Plateau. Lowercase letters indicate significant differences between treatments for each soil depth based on Tukey’s HSD pairwise comparisons.

**Data S1.** Supporting Information.

## Data Availability

Data used in the study are available in the Supporting Information.
