# Supplementary material for: Effects of Vegetation Restoration Type on Soil Greenhouse Gas Emissions and Associated Microbial Regulation on the Loess Plateau
Source: Ecol Evol. 2024 Dec 23;14(12):e70688. doi: 10.1002/ece3.70688 (PMC11664210; doi:10.1002/ece3.70688)
Supplement: Supplementary file 1 — Figure S1. Soil gram‐positive and gram‐negative bacterial PLFA abundance at different depths following five types of vegetation restoration on the Loess Plateau. Lowercase letters indicate significant differences between treatments for each soil depth based on Tukey’s HSD pairwise comparisons. [file ECE3-14-e70688-s001.docx]

**Supplementary materials**

**A**

**B**

**Fig. S1** Soil gram-positive and gram-negative bacterial PLFA abundance at different depths following five types of vegetation restoration on the Loess Plateau. Lowercase letters indicate significant differences between treatments for each soil depth based on Tukey^’^s HSD pairwise comparisons.

**Soil depth (cm)**

**Soil depth (cm)**
